# Ethnobotanical Study of Medicinal Plants Used by Traditional Healers to Treat Cancer-Like Symptoms in Eleven Districts, Ethiopia

**DOI:** 10.1155/2020/7683450

**Published:** 2020-04-21

**Authors:** Solomon Tesfaye, Anteneh Belete, Ephrem Engidawork, Teferi Gedif, Kaleab Asres

**Affiliations:** School of Pharmacy, College of Health Sciences, Addis Ababa University, Addis Ababa, Ethiopia

## Abstract

There is no ethnobotanical study conducted specifically on medicinal plants traditionally used to treat cancer in Ethiopia. Yet, traditional herbalists in different parts of the country claim that they have been treating cancer-like symptoms using herbal remedies. The objective of this study was to document medicinal plants traditionally used to treat cancer-like symptoms in eleven districts, Ethiopia. Traditional herbalists were interviewed using semistructured questionnaires, and field visits were also carried out to collect claimed plants for identification purpose. Seventy-four traditional herbalists, who claimed that they knew about and/or had used medicinal plants to treat cancer-like symptoms, were selected using the snowball method and interviewed. Herbalists used their intuition and relied on the chronicity, growth of external mass, and spreading of the disease to other parts of the body, as a means to characterize cancer symptoms. Furthermore, in some of the study districts, herbalists reported that they treat patients who had already been diagnosed in modern healthcare institutions prior to seeking help from them. The inventory of medicinal plants is summarized in a synoptic table, which contains the scientific and vernacular names of the plants, their geographical location, the parts of the plants, and the methods used to prepare the remedies. A total of 53 traditionally used anticancer plants, belonging to 30 families, were identified during the survey. The most frequently reported anticancer plants were *Acmella caulirhiza* Del (Asteraceae), *Clematis simensis* Fresen. (Ranunculaceae), *Croton macrostachyus* Del. (Euphorbiaceae), and *Dorstenia barnimiana* Schweinf. (Moraceae). Organizing traditional healers, documenting their indigenous knowledge, and scientifically validating it for the development of better cancer therapeutic agents constitute an urgent and important task for policymakers and scientists.

## 1. Introduction

Cancer is a complex disease that is very heterogenic and variable at cellular level and also differs from one patient to the other in its behaviour, development, and outcome [1]. Physical, metabolic, and behavioural variations of cancer cells from normal ones arise through the accumulation of genetic modifications and help them to proliferate rapidly, escape from host immune surveillance, and ultimately invade distant tissues [2]. Histopathological, genetic, and epigenetic and clinical outcome variations between and within different types of cancers have been the greatest challenge to understand the disease and develop novel therapies [3].

Surgery and radiation therapy were the most preferred means of treatment to control cancer before 1950 and after 1960, respectively [4]. Chemotherapy can be done before surgery to shrink the tumor or after surgery to kill the remaining cancer cells [5]. However, most of the chemotherapeutic drugs lack specificity and tend to rapidly damage normal dividing tissues, causing side effects such as immunosuppression, neurotoxicity, and hair loss [6]. Moreover, resistance has also reduced therapeutic efficacy of some anticancer chemotherapeutic drugs [7].

In order to address these limitations, tapping nature as a major source of chemically diverse novel anticancer compounds is a consistently proven track [8]. Screening natural products yield more hit with more “drug-like” characteristics (absorption and metabolism) as compared to screening of rationally designed compounds [9]. Furthermore, screening medicinal plants based on traditional use provides a higher chance of finding active plants relative to the random approach [10].

Ethiopia has a rich and diverse heritage of traditional medical practices, known for using plants to prepare more than 90% of the remedies [11]. In addition, the country has more than 6,500 higher plant species of which, around 12% are endemic [12]. Reports indicate that up to 80% of the population relies on traditional remedies as a primary source of health care [13]. Only few ethnobotanical reports from different agroecological zones of Ethiopia are available in the literature regarding medicinal plants used for cancer treatment. These include *Bersama abyssinica*, *Buddleja polystachya*, *Clerodendrum myricoides*, *Dovyalis abyssinica*, *Ekebergia capensis*, *Myrsine melanophloeos*, *Olea capensis*, *Pentas lanceolata*, *Sideroxylon oxyacanthum*, and *Zingiber officinale* [14]; *Bidens macroptera*, *Clematis simensis*, *Ferula communis*, and *Punica granatum* [15]; *Rumex abyssinicus* [16]; *Zanthoxylum chalybeum* [17]; *Phytolacca dodecandra* and *Vinca rosea* [18]; *Kalanchoe lanceolata*, *Stephania abyssinica*, and *Vernonia hymenolepis* [19]; *Plumbago zeylanica* [20–22]; *Acalypha acrogyna*, *Carissa spinarum*, *Maytenus ovatus*, and *Salvia nilotica* [23]; *Croton macrostachyus* [24]; and *Dorstenia barnimiana* [25, 26].

In view of this fact and considering the weak traditional recording and knowledge transfer system and an alarming rate of environmental degradation, finding anticancer plants and documenting their ethnobotanical information constitute an urgent and indispensable task. Therefore, the main aim of this study was to establish an inventory of medicinal plants traditionally used to treat cancer in eleven districts of Ethiopia.

## 2. Materials and Methods

### 2.1. Description of the Study Areas

**Figure 1:**
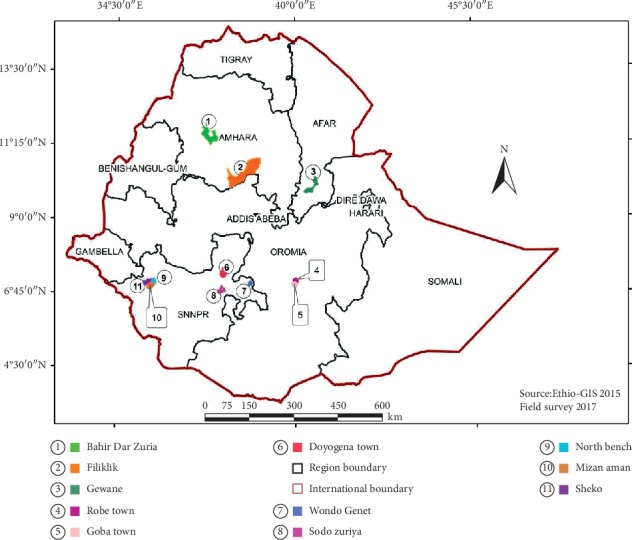
Map of Ethiopia showing the location of study districts.

This ethnobotanical study was conducted in four national regional states of Ethiopia: Oromia, Amhara, Afar, and Southern Nations, Nationalities, and People. The survey included different districts from each region, namely, Bale Robe and Goba from Oromia, Bahir Dar Zuria and Filiklik from Amhara, Gewane from Afar, and Wondo Genet, Sodo Zuria, Doyo Gena, North Bench, Mizan Aman, and Shako from Southern Nations, Nationalities, and People Regional State (Figure 1). These geographically, culturally, and agroecologically different study areas (Table 1) were selected mainly based on the availability of traditional healers and recommendations from health workers.

### 2.2. Data Collection

A team comprising a botanist and researchers from Addis Ababa University was set up, and health authorities were contacted for permission and identification of traditional herbalists living in each study area. Altogether, 117 traditional healers were approached using the snowball technique and 74 traditional healers who used herbs to manage cancer-like symptoms were selected. Ethnobotanical data were collected between January and August 2016, mainly through individual interviews with the selected traditional herbalists using a semistructured interview questionnaire. The questionnaire was prepared in Amharic language and translated to different local languages for traditional healers who do not speak Amharic. This questionnaire was designed to obtain information in the following areas: (i) general data on the informant, (ii) school attendance, (iii) use of plants for cancer treatment, (iv) source of the plant material, (v) part of the plant used, (vi) method of medicinal preparation, (vii) route of administration, and (viii) side effects.

A traditional healer for the purpose of this study is “a person who is recognized by the community in which s/he lives as competent to provide healthcare by using plants and plant products.” Each traditional healer was approached, briefed about the purpose of the research, and asked for his/her verbal consent in talking about cancer and its treatment. They were assured of the confidentiality of the information they provided. If plants were mentioned for their anticancer purposes, a botanical sample was collected. These specimens were pressed and preserved for later identification at the National Herbarium, Addis Ababa University, Addis Ababa, and a voucher specimen of each plant was deposited in the institute. All botanical names have been transcribed according to the nomenclature system used by the Plant List (http://www.theplantlist.org).

### 2.3. Data Analysis

The relative importance of medicinal plants used in the management of cancer-like symptoms in study areas was assessed using the relative frequency of citation (RFC), use value (UV), informants consensus factor (ICF), and cultural importance index (CI).

#### 2.3.1. Relative Frequency of Citation (RFC)

The RFC was calculated by dividing the number of informants that cite a particular plant species (FC) by the total number of informants in the survey (*N*) [29]:

(1) $$\begin{array}{c} \text{RFC}=\frac{\text{FC}}{N}. \end{array}$$

#### 2.3.2. Use Value (UV)

The use value demonstrates the relative importance of plant species to treat particular ailment, and it is determined by the following formula [30]:

(2) $$\begin{array}{c} \text{UV}=\sum \frac{U_{i}}{N_{i}}, \end{array}$$

where “UV” stands for the use value of a species, “*U*_*i*_” stands for the number of use reports cited by informants for that plant species, and “*N*_*i*_” is the total number of informers who reported the particular plant species *i*.

#### 2.3.3. Informant Consensus Factor (ICF)

Informant consensus factor (ICF) was calculated to determine the homogeneity of the information collected about particular plant species to treat specific ailment. It was estimated using the following formula [31]:

(3) $$\begin{array}{c} \text{ICF}=\frac{\text{Nur}-\text{Nt}}{\text{Nur}-1}, \end{array}$$

where Nur is the number of use reports of informants for particular ailment category and Nt refers to the number of species used for the ailment category by all informants.

#### 2.3.4. Cultural Importance Index (CI)

Cultural importance index (CI) is calculated by the sum of the use reports (UR) of informants mentioning each species use (from *i*_1_ to *i*_*N*_) in each use category and adding all the UR of each category (from *u*_1_ to *u*_NC_) divided by the total number of informants *N*. This index is determined by the following formula [29]:

(4) $$\begin{array}{c} {\text{CI}}_{i}\overset{u_{\text{NC}}}{\underset{u=u_{1}}{\sum }}\overset{i_{N}}{\underset{i=i_{1}}{\sum }}\frac{\text{UR}u_{i}}{N}, \end{array}$$

where CI is an ethnobotanical index that indicates the spread of the use along with the diversity of uses of each species.

## 3. Results

**Figure 2:**
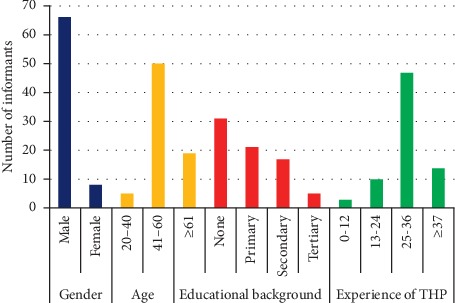
Demographic details of the interviewed informants.

The informants consisted of 66 male and 8 female traditional healers and they were divided into three age groups: 20–40, 41–60, and ≥ 61 years. Out of 74 interviewed traditional healers, most of them (*N*%) were adults aged between 41 and 60 years. Majority of the respondents (70.2%) gained their knowledge from family members and 82% of all interviewed respondents practiced ethnomedicine for more than 25 years. More than 70% of the respondents were either only at their primary level of education or did not have a formal education at all (Figure 2). Traditional healers usually used their intuition and relied on the chronicity and growth of external mass, as a means to diagnose cancer. Lumpy growth was the most commonly cited criteria used to diagnose cancer, followed by ulcerative wounds and bleeding (Table 2). However, there were instances where some of the healers claimed to have treated patients already diagnosed with cancer at modern health institutions. Traditional healers identified cancer as “*Nekersa*” in Bahir Dar Zuria and Filiklik, “*Naqarsa*” in Bale Robe and Goba, “*Sissac*” in Gewane, “*Xoka* or *Toka*” in Doyo Gena, “*Balamo*” in Wondo Genet, “*Kums* or *niamt*” in North Bench, and “*Kanser*” in Sheko and Sodo Zuria district. Out of the 6 specific cancer types (skin, breast, lung, cervical, throat, and intestinal) claimed to be treated by the respondents, skin cancer was a dominant one followed by breast cancer.

**Figure 3:**
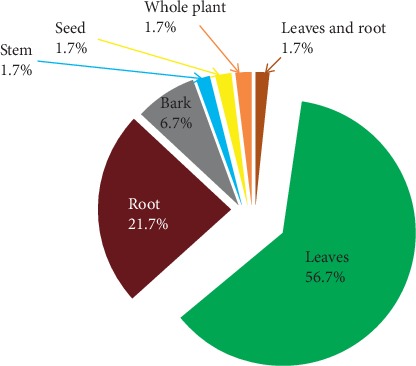
Frequency of plant parts used for the preparation of medicinal remedy.

A total of 53 plant species belonging to 30 families were reported for their anticancer use (Table 3). The result of this study showed that shrubs (49.1%), herbs (33.9%), trees (13.2%), and climbers (3.8%) were the main sources of potential anticancer medicinal plants. This study also indicated that leaves (56.7%) were the most commonly used plant parts followed by roots (21.7%), bark (6.7%), stem (1.7%), seeds (1.7%), whole plant (1.7%), leaves and roots (5%), leaves or stem (1.7%), and leaves or seeds (1.7%) (Figure 3). Most of the reported plants occurred naturally in wild (96.2%); however, cultivation was also a source (3.8%). Reported medicinal plants have been traditionally claimed to be used to treat different types of ailments including cancer. However, only few have been scientifically investigated for their antiproliferative or cytotoxic activity (Table 4). While comparing the amount and distribution of anticancer plants in the past ten years, regardless of the study areas, all respondents believed that the amount and distribution of these plants are reduced.

In the current study, the highest UVs were recorded for *Aloe* spp. (6), *Albizia schimperiana* (4), *Sida schimperiana* (4), *Achyranthes aspera* (4), *Brucea antidysenterica* (4), *Cleome brachycarpa* (3), *Leonotis ocymifolia* (3), and *Prunus africana* (3). The lowest UVs were obtained for *Acokanthera schimperi*, *Acmella caulirhiza*, *Cineraria abyssinica*, and *Gnidia involucrata* (Table 3). A total of 228 use reports have been documented and categorized into seven categories (Table 5). Among these, other ailments (46.3%) and skin cancer (26.5%) had the highest use reports. Furthermore, ICF values were also calculated and ranged from 0 to 0.42. The highest ICF values were recorded in other ailments (0.42) and breast cancer (0.32) followed by skin cancer (0.23) category (Table 5). The other ailments category comprises of diseases such as stomach ache, malaria, wart, swelling, wounds, evil eye, toothache, bleeding, gastrointestinal disorder, headache, bone fracture, cough, snake bite, herpes simplex, tonsillitis, hypertension, dandruff, fever, and hemorrhoid. The ICF value of the remaining four categories (lung cancer, colon cancer, cervical cancer, and throat cancer) was zero. Quantitative ethnobotanical indexes such as RFC and CI were calculated in this study to analyze the ethnobotanical information. According to RFC values, *Croton macrostachyus* (0.1), *Vernonia auriculifera* (0.04), *Clematis simensis* (0.04), and *Acmella caulirhiza* (0.04) are the most frequently cited among all reported plants. *Croton macrostachyus* (0.16), *Dorstenia barnimiana* (0.12), and *Aloe* spp. (0.08) rank 1^st^, 2^nd^, and 3^rd^ in position, respectively, according to the CI reference. Our result also shows that the Pearson correlation coefficient of RFC was positively and negatively correlated to CI and UV, respectively (Table 6).

Most of the reported remedies, prepared from these plants, were either applied topically (50%) or taken orally (41.7%). The remaining remedies were prepared to be administered either topically or orally (3.3%), both topically and orally (1.7%), and intranasally (1.7%). Usually, fresh plants were finely chopped, dried, and pounded to powder form. Then, the powder of either one or the combination of more than one plant was either mixed with drinking water or pasted and applied topically. In other cases, fresh plant parts were decocted and taken orally or crushed and applied topically. Water was the main medium in preparation of most remedies and additives like honey, milk, and butter were also used. To determine the amount of plant parts used to prepare remedies, traditional healers used spoon, fingertip, and number (in case of fresh leaves). Adverse effects reported by respondents include vomiting, diarrhea, and skin ulcers.

## 4. Discussion

Despite the rich biodiversity of the study areas, broad acceptability, and centuries-old tradition of using traditional medicines, the number of anticancer plants reported in this study is far less than expected. As it was reported by different ethnobotanical studies conducted in different parts of Ethiopia, this could be attributed to the attitude of many traditional healers to guard their indigenous medical knowledge as a family secret and hence hesitant to share with the researchers [13, 32, 73]. Justifying the lower number of female traditional healers (8, 11%) participated in this study, these studies also inferred that traditional healers usually pass their knowledge to the first son of the family.

In this study, in agreement with the studies conducted in Fiche district [35], Ghimbi district [20], and Hawassa city [17] of Ethiopia, the predominant botanical families recorded, listing over 5 plant species each, were Asteraceae, Fabaceae, and Lamiaceae. This could be due to the fact that these families are the largest in the flora of Ethiopia and Eritrea [15, 21, 143]. Moreover, cytotoxicity studies conducted on different Mexican plants reported that the highest number of plant species with both *in vitro* and *in vivo* antineoplasic activities was from these families [20].

The highest UVs recorded in this study include *Aloe* spp. (6), *Achyranthes aspera* L. (4), *Albizia schimperiana* (4), *Sida schimperiana* (4), and *Brucea antidysenterica* (4). The highest ICF value (0.42) recorded for “other ailments” category, in this study, suggests that informants are in agreement with the use of particular plant species to treat ailments in this category. The lowest ICF value (0) obtained was for lung, colon, cervical, and throat cancer categories. This might be due to the cultural and ecological differences of the study sites and the difficulty to pinpoint the physical symptoms of lung, colon, cervical, and throat cancer as compared to the breast and skin cancer.

The present study also revealed that RFC and CI values of some reported species are similar. However, there is a distinct difference in species ranking using each index. *C. macrostachyus* is placed in the first position according to both RFC and CI index. This could be due to the fact that this species is mentioned by many informants and is the most recognized plant in most study areas. Furthermore, CI value of *C. macrostachyus* is also high, suggesting the diversified use of the plant. *V. auriculifera* and *C. simensis* ranked next to *C. macrostachyus*, according to RFC index. On the other hand, *D. barnimiana* and *Aloe* spp. ranked 2^nd^ and 3^rd^ by CI index. It has been suggested that UV value is a good measure of use diversity, than the number of citations [144]. In agreement with this, UV value in our study is driven by species with greatest number of use rather than those cited by more informants. The Pearson correlation coefficient of −0.36, between RFC and UV, shows significant negative association between the local importance of each medicinal plant and relative importance of use of plants. This result is in contrast to previous studies that reported a significant positive correlation between RFC and UV [145, 146]. On the other hand, there is a significant positive correlation between RFC and CI (*r*^2^ = 0.74, *p* < 0.001) implying that their pattern matches across species. The species with larger RFC value usually have higher CI, such as *Croton macrostachyus* and *Vernonia auriculifera*.

Leaves and roots are the most commonly used plant parts in the preparation of remedies in the study districts. Similarly, other ethnobotanical studies conducted in different parts of Ethiopia also reported that leaves are the dominant plant part followed by root [16–18, 20]. The preference towards leaves may be because leaves are the main photosynthetic organs in plants and the primary reservoirs for secondary metabolites with medicinal values [36]. In contrary to other ethnobotanical studies [17, 18], where the common use of concoctions and oral route were reported, in the current study majority of the reported remedies are prepared from a single plant species and applied topically.

Comparative analysis of this study with other ethnobotanical surveys of plants used traditionally in treating and managing cancer in Ethiopia [18], Kenya [147], Cameroon [37], Nigeria [19, 38], South Africa [39], and Bangladesh [148] revealed some similarities in the plants cited in these surveys. Of the 30 plant species cited to be used in Ethiopia [18], 7 species are identified in our study: *Bersama abyssinica* Fresen., *Brucea antidysenterica* JF. Mill., *Calpurnia aurea* (Ait.) Benth. *Dodonaea angustifolia* L.f., Dorstenia barnimiana Schweinf, *Kalanchoe petitiana* A. Rich., and *Prunus africana* (Hook. f) Kalkm.

Although herbal remedies are believed by the general public to be safe [46], some research findings suggested otherwise. For instance, traditionally used Thai anticancer plants *Ganoderma lucidum* (Fr.) Karst., *Houttuynia cordata* Thunb., and *Saussurea involucrata* Matsum. & Koidz. were reported to cause side effects such as headache, insomnia, constipation, and diarrhea [62]. Similarly, side effects such as vomiting, diarrhea, and skin necrosis, associated with the use of traditional herbal remedies, were reported in this and other ethnobotanical studies conducted in Ethiopia [149, 150]. Few side effects reported in this study, as compared to other ethnobotanical studies conducted in Ethiopia, could be attributed to the frequent use of the topical route of administration. Nevertheless, considering the probability of underreporting adverse effects, extensive toxicological investigations should be conducted to protect the public.

*In vitro* cytotoxicity and antioxidant properties of some of the plants reported in our study have also been studied. Among these plants, potent cytotoxic activity was reported for knipholone anthrone isolated from *Kniphofia foliosa*, with IC_50_ value that ranges between 0.9 ± 0.1 and 3.3 ± 0.4 *μ*g/mL [89]. Similarly, Nibret and Wink reported the cytotoxic activity of the crude extract of *Acokanthera schimperi* with IC_50_ value of 7.1 *μ*g/mL [73]. Studies conducted on the leaves of *Cineraria abyssinica* [100], bark of *Senna singueana* [116], and bark and leaves of *Rumex nepalensis* [79] also revealed potent radical scavenging activity of these plants.

## 5. Conclusion

The present study showed that traditional healers in eleven districts of Ethiopia use different medicinal plants to manage cancer-like symptoms. Frequency of citation value ranked *Croton macrostachyus* Del., *Clematis simensis* Fresen., *Dorstenia barnimiana* Schweinf, *Vernonia auriculifera* Hiern, and *Acmella caulirhiza* Del. as most cited plant species in study areas. Hence, based on these findings, we are currently evaluating the *in vitro* antiproliferative activities of reported medicinal plant species with a higher frequency of citation against human breast adenocarcinoma (MCF-7), human uterine cervical adenocarcinoma (SiSo), human lung carcinoma (A-427), and human bladder cancer (RT-4) cell lines using crystal violate assay. However, considering the rapid disappearance of the traditional knowledge of medicinal plants and an urgent need for new anticancer agents, additional studies have to be conducted to document and scientifically validate traditionally used Ethiopian anticancer plants.

## Figures and Tables

**Table 1:** Vegetation type, climatic condition, and demographic data of the study areas [27, 28] (source: National Meteorological Service Agency of Ethiopia).

District	Distance from capital city (km)	Approximate population (2015)	Number of interviewed healers	Area size (km^2^)	Geographical location	Average elevation above sea level (m.a.s.l)	Vegetation type	Climatic condition (2014)	
								Annual average rainfall (mm)	Annual average temperature range (°C)
Bale Robe	432	65,284	2	8.87	7°07′11.65″ N 40°00′24.82″ E	2480	DAF	745.6	9.2–23.2
Goba	444	47,135	7	20.15	7°00′41.66″ N 39°58′33.96″ E	2614	DAF	736.3	9.5–23.8
Bahir Dar Zuria	578	206,708	16	1443.37	11°34′27.15″ N 37°21′40.87″ E	1800	CTW, DAF, and FLV/MFS	1547.1	12.7–27.6
Filiklik	188	142,722	7	806.98	10°02′12.74″ N 38°14′27.65″ E	1853	CTW and DAF	880.2	12.9–22.0
Gewane	344	39,186	6	967.85	10°29′59.99″ N 40°44′59.99″ E	568	ACB	586.7	19.5–36.7
Wondo Genet	270	196,277	12	226.45	7°05′3.01″ N 38°37′8.02″ E	1742	DAF	928.7	15–29.6
Sodo Zuria	383	145,092	2	25.62	6°51′10.11″ N 37°45′39.49″ E	1854	CTW and DAF	1569.2	14.8–25.2
Doyo Gena	258	95,393	14	130.57	7°21′20.22″ N 37°47′07.15″ E	2300	DAF	1334.5	11–22.8
North Bench	587	126,308	4	392.65	6°37′53.43″ N 35°33′56.83″ E	2367	CTW	1671.8	16–33.3
Mizan Aman	565	64,996	3	24.45	6°59′37.13″ N 35°34′55.92″ E	1441	CTW and MAF	1963.7	14.8–28.8
Shako	617	51,195	1	48,089.63 ha	7°33′42.37″ N 35°39′11.83″ E	1800	CTW and MAF	1906.9	11.4–22.4

*Note*. Vegetation type: DAF: dry evergreen Afromontane forest and grassland complex; CTW: Combretum-Terminalia woodland and wooded grassland; FLV/MFS: freshwater marshes and swamps, floodplains, and lake shore vegetation; ACB: Acacia-Commiphora woodland and bushland proper; MAF: moist evergreen Afromontane forest. m.a.s.l: meter above sea level; mm: millimeter; °C: degree Celsius; km^2^: kilometer square.

**Table 2:** Symptoms that are used by traditional healers to diagnose cancer.

Cancer types	Reported symptoms	Number of traditional healers
Skin	Lumpy growth	32
	Spreading pea-sized growth	1
	Ulcerative growth and oozing blood	1

Breast	Lumpy growth	17
	Lumpy growth on one breast and progressive weight loss	1
	Ulcerative wounds on breast	5
	Ulcerative wounds on breast and swelling on armpit and neck	1
	The patient was receiving anticancer treatment for breast cancer in hospital	12

Cervical	Foul-smelling bloody vaginal discharge, pain during sexual intercourse, and weight loss	1

Colon	Chronic rectal bleeding and weight loss	1

Lung	Coughing up blood	1

Throat	Coughing and swelling on the neck	1

**Table 3:** List of candidate medicinal plants traditionally used for cancer treatment in the study areas.

Voucher number	Botanical name (family)	Vernacular name	Districts	Growth form	Habitat	Parts used	Preparation	Type of cancer treated	Application	UV	RFC	CI
	Acanthaceae											

Bele-047	*Justicia schimperiana* (Hochst. ex Nees) T. Anderson	*Kitkit*	North Bench	Shrub	Wild	Roots	Fresh roots are crashed and boiled, and the cool decoction is drunk before meal	Lung	Oral	1	0.027	0.067
Bele-057	*Justicia schimperiana* (Hochst. ex Nees) T. Anderson	*Gulbana*	Doyo Gena	Shrub	Wild	Leaves	Fresh leaves are pounded, and the juice is applied on the affected area	Skin	Topical			

	Aloaceae											

Bele-060	*Aloe* sp.	*Gurta waqota*	Doyo Gena	Shrub	Wild	Leaves	Fresh roots are crashed, and the sap is applied on the affected area	Skin	Topical	6	0.014	0.081

	Amaranthaceae											

Bele-044	*Achyranthes aspera* L.	*Koch ashite*	Mizan Aman	Herb	Wild	Leaves	Leaves are roasted on metal plate, pounded into powder, mixed with animal butter, and smeared on the affected part	Skin	Topical	4	0.014	0.054

	Apiaceae											

Bel-046	*Centella asiatica* (L.) Urb.	*Gorongoch*	Sheko	Herb	Wild	Leaves	Young leaves are crashed, and the sap sniffed	Throat	Intranasal	2	0.014	0.027
Bel-002	*Hydrocotyle mannii* Hook.f	*Ye'ti medhanit*	North Bench	Herb	Wild	Leaves	Young leaves are crashed and applied on the affected area	Skin	Topical	1	0.014	0.014

	Apocynaceae											

Bel-003	*Acokanthera schimperi* (A.DC.) Schweinf.	*Merenz*	Bahir Dar Zuria	Shrub	Wild	Leaves	Young leaves are crashed and applied	Skin	Topical	0.5	0.027	0.027
Bel-009	*Carissa spinarum* L.	*Agam*	Bahir Dar Zuria	Shrub	Wild	Leaves	Leaves are crashed and infused in cold water overnight and drunk before meal and applied on the affected area	Skin	Oral	1	0.027	0.027

	Asclepiadaceae											

Bel-040	*Calotropis procera* (Aiton) Dryand.	*Qumbo*	Gewane	Shrub	Wild	Roots	Fresh roots are crashed, and the sap is applied on the affected area	Breast	Topical	3	0.014	0.027
Bel-036	*Pentarrhinum insipidum* E. Mey.	*Barohula*	Gewane	Shrub	Wild	Roots	Fresh roots are crashed, and the sap is applied on the affected area	Breast and skin	Topical	1	0.014	0.014
Bel-037	*Echidnopsis dammanniana* Sprenger	*Mureli*	Gewane	Herb	Wild	Stem	Stems are cut, and the sap is applied	Skin	Topical	2	0.014	0.027

	Asphodelaceae											

Bel-020	*Kniphofia foliosa* Hochst.	*Shushube*	Bale Goba	Shrub	Wild	Roots	Dry roots are pounded, and the powder is mixed with honey	Cervical and breast	Oral	1	0.027	0.027
	Asteraceae											
Bel-045	*Acmella caulirhiza* Delile	*Kust asht*	Mizan Aman	Shrub	Wild	Leaves	Young leaves are chewed by the healer and spit on	Breast	Topical	0.67	0.04	0.054
Bel-049	*Acmella caulirhiza* Delile	*Bitisa*	Wondo Genet	Shrub	Wild	Leaves	Fresh leaves are crashed and infused in cold water	Breast	Oral			
Bel-030	*Artemisia absinthium* L.	*Natrara*	Sodo Zuria	Herb	Wild	Leaves	Dried leaves are ground and macerated in coffee or tea	Breast	Oral	2	0.014	0.027
Bel-029	*Artemisia afra* Jacq. ex Willd.	*Agufa*	Doyo Gena	Herb	Wild	Leaves	Dried leaves are ground and macerated in coffee or tea	Breast	Oral	1	0.014	0.014
Bel-031	*Artemisia annua* L.	*Artemisia*	Sodo Zuria	Tree	Cultivated	Leaves	Dried leaves will be ground and decocted in hot water	Breast	Oral	1	0.014	0.014
Bel-021	*Cineraria abyssinica* Sch.Bip. ex A.Rich.	Unknown	Bale Robe	Herb	Wild	Leaves	Fresh leaves are pounded, and the sap is applied on the affected area	Skin	Topical	1.5	0.027	0.054
Bel-058	*Guizotia scabra* (Vis.) Chiov.	*Sheshota*	Doyo Gena	Shrub	Wild	Leaves	Fresh leaves are pounded, and the sap is applied on the affected area	Skin	Topical	1	0.014	0.014
Bel-034	*Solanecio gigas* (Vatke) C. Jeffrey	*Arbaba*	Doyo Gena	Shrub	Wild	Leaves	Fresh leaves are pounded and the sap is applied on the affected area	Skin	Topical	2	0.014	0.027
Bel-025	*Vernonia auriculifera* Hiern	*Barawa*	Doyo Gena	Shrub	Wild	Leaves	Fresh leaves are pounded, and the sap is applied on the affected area	Skin	Topical	1.33	0.041	0.081
Bel-056	*Vernonia auriculifera* Hiern	*Reji*	Wondo Genet	Shrub	Wild	Leaves	Fresh leaves are chewed by the healer and spit on	Skin	Topical			

	Capparidaceae											

Bel-039	*Cleome brachycarpa* (Forssk.) Vahl ex DC.	*Berbere*	Gewane	Herb	Wild	Leaves	Fresh leaves are pounded, and the sap is applied on the affected area	Breast and skin	Topical	3	0.014	0.014

	Commelinaceae											

Bel-026	*Commelina benghalensis* L.	*Laluncha*	Doyo Gena	Herb	Cultivated	Roots	Fresh roots are pounded, and the sap is applied on the affected area	Skin	Topical	2	0.014	0.027

	Crassulaceae											

Bel-019	*Kalanchoe petitiana* A. Rich.	*Anchura*	Bale Goba	Shrub	Wild	Leaves	Fresh leaves are roasted for 2 minutes and applied on the affected area	Breast and skin	Topical	1.5	0.027	0.041

	Euphorbiaceae											

Bel-012	*Croton macrostachyus* Hochst. ex Delile	*Bisana*	Filiklik	Tree	Wild	Leaves or stem	Fresh leaves or succulent stems are crashed, and the sap is applied on the affected area	Breast and skin	Topical	0.75	0.1	0.16
Bel-035	*Croton macrostachyus* Hochst. ex Delile	*Besena*	Doyo Gena	Tree	Wild	Bark	Dry bark is pounded, and the powder is applied on the affected area	Skin	Topical			
Bel-048	*Croton macrostachyus* Hochst. ex Delile	*Masichoo*	Wondo Genet	Tree	Wild	Leaves	Fresh leaves are crashed, macerated in cold water, and drunk	Breast and skin	Oral			
Bel-032	*Euphorbia schimperiana* Scheele	*Gendalelata*	Doyo Gena	Shrub	Wild	Roots	Fresh roots are pounded, and the sap is applied on the affected area	Skin	Topical	1	0.014	0.014

	Fabaceae											

Bel-014	*Albizia schimperiana* Oliv.	*Sessa*	Filiklik	Tree	Wild	Leaves	The mixture of fresh leaves of *Albizia schimperiana* and *Carissa spinarum* is macerated in cold water for 2 days, and the macerated liquid is drunk	Breast, intestinal, and skin	Oral	4	0.014	0.014
Bel-004	*Calpurnia aurea* (Aiton) Benth.	*Digita*	Bahir Dar Zuria	Shrub	Wild	Leaves or seeds	Dry leaves or seeds are ground, macerated in cold water, and drunk	Breast	Oral	2	0.014	0.027
Bel-023	*Crotalaria agatiflora* Schweinf.	Unknown	Bale Goba	Shrub	Wild	Seeds	Dry seeds are ground, mixed with honey, and applied	Skin	Topical	1	0.014	0.014
Bel-028	*Crotalaria incana* L.	*Chelke*	Doyo Gena	Shrub	Wild	Leaves	Fresh leaves are crashed, and the sap is applied on the affected area	Skin	Topical	1	0.014	0.014
Bel-007	*Senna singueana* (Delile) Lock	*Gefa*	Bahir Dar Zuria	Shrub	Wild	Leaves	Fresh leaves are crashed, macerated, and drunk	Skin	Oral	2	0.014	0.027

	Lamiaceae											

Bel-043	*Ajuga leucantha* Lukhoba	*Tiks asht*	North Bench	Herb	Wild	Leaves	Fresh leaves are crushed, and the sap is applied on the affected area	Breast	Topical	1	0.014	0.014
Bel-024	*Leonotis ocymifolia* (Burm.f.) Iwarsson	*Armagusa*	Bale Goba	Herb	Wild	Leaves	Fresh leaves are crashed, macerated overnight, and drunk	Breast and skin	Oral	3	0.014	0.014
Bel-054	*Ocimum gratissimum* L.	*Mekedesisa*	Wondo Genet	Herb	Wild	Roots	Fresh roots are crushed, boiled, and drunk	Skin	Oral	2	0.014	0.027
Bel-059	*Pycnostachys abyssinica* Fresen.	*Tontona*	Doyo Gena	Herb	Wild	Leaves	Fresh leaves are crushed, and the sap is applied on the affected area	Skin	Topical	2	0.014	0.027
Bel-042	*Salvia nilotica* Juss. ex Jacq.	*Barnbanch*	North Bench	Shrub	Wild	Whole plant	Dry plant parts are ground, mixed with honey, and applied	Breast	Topical	2	0.014	0.027
Bel-022	*Thymus schimperi* Ronniger	*Tosigne*	Bale Goba	Herb	Wild	Leaves	Dry leaves are decocted and drunk	Breast	Oral	2	0.014	0.027

	Malvaceae											

Bel-051	*Sida schimperiana* Hochst. ex A. Rich.	*Kotijebessa*	Wondo Genet	Shrub	Wild	Roots and leaves	Fresh leaves and roots are crashed, macerated, and drunk	Breast and skin	Oral	4	0.014	0.027

	Melianthaceae											

Bel-001	*Bersama abyssinica* Fresen.	*Azamir*	Bahir Dar Zuria	Shrub	Wild	Bark	Dry bark is ground, macerated, and drunk before meal	Breast	Oral	1	0.014	0.014

	Moraceae											

Bel-008	*Dorstenia barnimiana* Schweinf.	*Work Bemeda*	Bahir Dar Zuria	Herb	Wild	Roots	Dry roots are ground, mixed with water and honey, and drunk, or dry roots are ground, mixed with honey, and applied on the affected area	Breast	Oral or topical	0.6	0.068	0.12

	Myrtaceae											

Bel-006	*Syzygium guineense* (Willd.) DC.	*Dokima*	Bahir Dar Zuria	Tree	Wild	Leaves and roots	Dry leaves and roots of *Syzygium guineense* and dry leaves of *Osyris quadripartita* are ground, mixed, decocted, and drunk	Skin	Oral	2	0.014	0.027

	Oxalidaceae											

Bel-052	*Oxalis corniculata* L.	*Qinta*	Wondo Genet	Herb	Wild	Leaves and roots	Fresh leaves and roots are crashed and applied with a bandage	Breast	Topical	2	0.014	0.027

	Polygonaceae											

Bel-018	*Rumex nervosus* Vahl	*Emboacho*	Filiklik	Shrub	Wild	Roots	Dry roots are ground, macerated, and drunk	Skin	Oral	3	0.014	0.041
Bel-033	*Rumex nepalensis* Spreng.	*Goecho*	Doyo Gena	Herb	Wild	Roots	Dry roots are ground and taken with food	Colon	Oral	1.5	0.027	0.041
Bel-053	*Rumex nepalensis* Spreng.	*Sharibicho*	Wondo Genet	Herb	Wild	Bark	Fresh bark is crashed and squeezed, and the sap is applied	Skin	Topical			

	Ranunculaceae											

Bel-010	*Clematis simensis* Fresen.	*Yeazo Hareg*	Bahir Dar Zuria	Climber	Wild	Leaves	Fresh roots of *Dorstenia barnimiana* mixed with fresh leaves of *Clematis simensis*, pounded, and applied	Breast	Topical	0.67	0.041	0.054

	Rosaceae											

Bel-011	*Prunus africana* (Hook.f.) Kalkman	*Tikur enchet*	Bahir Dar Zuria	Tree	Wild	Bark	Dry bark is ground, decocted, and drunk	Breast and skin	Oral	3	0.014	0.014
	Rutaceae											
Bel-016	*Clausena anisata* (Willd.) Hook.f. ex Benth.	*Limich*	Filiklik	Shrub	Wild	Leaves	Dry leaves are ground, mixed with honey, and eaten	Breast	Oral	2	0.014	0.027

	Santalaceae											

Bel-013	*Osyris quadripartita* Salzm. ex Decne.	*Keret*	Filiklik	Shrub	Wild	Leaves	Dry leaves are ground, decocted, and drunk	Breast	Oral	2	0.027	0.027

	Sapindaceae											

Bel-005	*Dodonaea viscosa* subsp. *angustifolia* (L.f.) J.G.West	*Kitkita*	Bahir Dar Zuria	Tree	Wild	Roots	Dry roots are ground, mixed with honey, and applied or dry roots are ground, decocted, and drunk	Breast, skin and cervical	Topical or oral	1	0.014	0.041

	Simaroubaceae											

Bel-017	*Brucea antidysenterica* J.F.Mill.	*Abalo*	Filiklik	Tree	Wild	Leaves	Dry leaves are ground, pasted with cold water, and applied	Skin	Topical	4	0.014	0.054

	Solanaceae											

Bel-027	*Discopodium penninervium* Hochst.	*Chechanga*	Doyo Gena	Shrub	Wild	Leaves	Fresh leaves are crashed and applied	Skin	Topical	1	0.014	0.014

	Thymelaeaceae											

Bel-055	*Gnidia involucrata* Steud. ex A.Rich.	*Bito*	Bahir Dar Zuria	Herb	Wild	Roots	Dry roots are ground, mixed with honey, and eaten	Breast	Oral	0.5	0.027	0.027

	Verbenaceae											

Bel-050	*Lantana trifolia* L.	*Hanshebello*	Wondo Genet	Shrub	Wild	Leaves	Fresh leaves are ground, macerated in cold spring water, and drunk	Breast and skin	Oral	2	0.014	0.014
Bel-015	*Lippia adoensis* Hochst.	*Kessie*	Filiklik	Shrub	Wild	Leaves	Dry leaves are ground, macerated in cold water, and drunk	Skin	Oral	2	0.014	0.027

	Vitaceae											

Bel-038	*Cyphostemma serpens* (Hochst. ex A.Rich.) Desc.	*Eiriti*	Gewane	Climber	Wild	Roots	Dry roots are ground, pasted with honey and eaten, and applied	Skin	Oral and topical	1	0.014	0.014

UV = use value; RFC = relative frequency of citation; CI = cultural importance index.

**Table 4:** Cross-reference of cancer treatment candidate plant species collected from the study areas with the published literature.

Botanical name (family)	Biological activity/chemical constituents	Illnesses/symptoms claimed to be treated traditionally
*Justicia schimperiana* (Hochst. ex Nees) T. Anderson (Acanthaceae)	Saponins, alkaloids, terpenoids and flavonoids [32] *In vitro* cytotoxicity [33]; *in vitro* antioxidant activity on DPPH assay [34]; *in vivo* suppression of parasitaemia on *Plasmodium berghei*-infected mice in the 4-day suppressive test [32]; and *in vivo* hepatoprotective activity in mice intoxicated with CCL_4_ [35]	Wound [15, 21]; rabies [15, 18–20, 36–39]; jaundice [15, 16, 21, 23, 38, 40]; gonorrhea [17, 36, 39]; liver cirrhosis [18, 26]; seizure [19, 41]; stomach ache [15, 25, 38]; helminths [15, 42, 43]; skin burn/lesion [23, 44]; arthritis [21, 23]; hepatitis [45, 46]; evil eye [15, 46]; dysentery [15, 21]; malaria [36, 39]; common cold, asthma, and headache [36, 39, 47]; otitis [48]; toothache [49]; and rheumatism [50]

*Aloe* sp. (Aloaceae)	Anthrones and chromones [51], pyrones, coumarins, alkaloids, glycoproteins, naphthalenes, and flavonoids [52] 7‐O‐methylaloeresin showed *in vitro* antioxidant activity in DPPH assay [51], and methanol and ethanol extract showed *in vivo* parasitaemia suppression on *Plasmodium berghei*-infected mice in the 4-day suppressive test [53, 54]	Wound [21, 55]; eye disease [21, 46, 48, 56]; snake bite [21, 48, 56]; malaria [20, 21, 44, 48, 54]; easing labour [44]; tropical ulcer, colon cleaner, and gallstone [48]; amoeba, abdominal pain, impotence, and urine retention [21]; dandruff [46, 56], hemorrhoids and hepatitis B [46]; ascariasis [21]; diabetes [54]; asthma [55]; foot strain [57, 58]; wart and anthrax [20]; external injury [59]; and liver swelling, splenomegaly, and skin inflammation [56]

*Achyranthes aspera* L. (Amaranthaceae)	Phytosteroids, polyphenols, and saponins [60] Methanol extracts have showed *in vivo* wound healing activity [61]	Bleeding [21, 24, 26, 62–64]; retained placenta [21, 62]; stomach ache and external swelling [17]; rhesus factor incompatibility in pregnancy [40, 55]; epistaxis [19]; hepatitis and evil eye [24]; tonsillitis [21, 57]; snake bite and paralysis [21]; dysentery [59]; herpes zoster [26]; anthrax [21, 49]; nasal infection and ophthalmic infection [64]; excessive menstruation and tape worm infection [15]; and gonorrhea [65]

*Centella asiatica* (L.) Urb. (Apiaceae)	Terpenoids (triterpenes, asiaticoside, centelloside, madecassoside, brahmoside, brahminoside (saponin glycosides), asiaticentoic acid, centellic acid, centoic acid, madecassic acid, terminolic acid, betulic acid, *β*-caryophyllene, trans-*β*-farnesene and germacrene D (sesquiterpenes), *α*-pinene, and *β*-pinene [66, 67] Methanol extract inhibited the proliferation of human gastric adenocarcinoma (MK-1), human uterine carcinoma (HeLa), and murine melanoma (B16F10) cells *in vitro* [68]; aqueous extracts induced apoptosis in colonic crypts and exerted chemopreventive effect on colon tumorigenesis in male F344 rats [69]	Genital infection and lymphadenitis [63]; topical swelling [26, 70]; gastritis, headache, and evil eye [70]; bleeding [40]; wound [24]; abdominal ache [71]; meningitis [72]; and tinea corporis [47]

*Hydrocotyle mannii* Hook.f (Apiaceae)	No previous reports	Eye infection [63] and cataract [72]
*Acokanthera schimperi* (A.DC.) Schweinf. (Apocynaceae)	*In vitro* cytotoxicity [73]; *in vitro* antiviral activity against coxsackie B3, influenza A, and herpes simplex type1 virus [74]; *in vitro* antimicrobial activity against *Staphylococcus aureus*, *Pseudomonas aeruginosa, Trichophyton mentagrophytes* [75]; and *in vivo* parasitaemia suppression in *Plasmodium berghei*-infected mice [76]	Wound [16, 44, 77, 78]; hepatitis [15, 16, 22, 44]; gonorrhea [19, 25]; evil eye [62]; bone fracture [24]; hemorrhoids [44]; scabies [21]; malaria and tonsillitis [48, 56]; psychiatric disease [55]; and skin diseases [65]

*Carissa spinarum* L. (Apocynaceae)	*In vitro* antioxidant activity on DPPH assay and *in* antiproliferative activity [79]	Throat cancer [23, 80]; evil eye [16, 21, 24, 49, 62, 70, 72, 81]; snake bite [23, 80]; gonorrhea [20, 65]; stomach ache [20, 70]; impotence and headache [20]; tonsillitis [17, 56, 70]; wound and febrile illness [16]; bleeding after delivery [44]; muscle cramps [49]; toothache [47]; and premature ejaculation [56]

*Calotropis procera* (Aiton) Dryand. (Asclepiadaceae)	Latex contains phytochemicals such as alkaloids, sterols, fatty acids, starches, sugars, oils, tannins, resins, and gums, and enzymatic proteins such as proteases, chitenases, lipases, peptidases, esterase, peroxidases, papain, hevein, and lectins [82] *In vivo* hepatoprotective [83]; hypoglycemic effect [84]; strong anti-implantation (antifertility) [85]; crude latex showed antioxidant and antiapoptotic activities against the toxicity of 4-nonylphenol [86]	Wound [16, 21, 81]; hemorrhoids [16, 19, 44]; wart [16, 57]; snake bite [23, 87]; kidney stone, tuberculosis, and scabies [16]; swelling [58]; skin rash [21, 49]; tinea capitis [21]

*Pentarrhinum insipidum* E. Mey. (Asclepiadaceae)	No previous reports	

*Echidnopsis dammanniana* Sprenger (Asclepiadaceae)	No previous reports	Snake bite [56]

*Kniphofia foliosa* Hochst. (Asphodelaceae)	2-Acetyl-1-hydroxy-8-methoxy-3-methylnaphthalene, 10-(chrysophanol-7′-yl)-10-(ξ)-hydroxychrysophanol-9-anthrone, chryslandicin, knipholone, and chrysophanol [88] 10-(Chrysophanol-7′-yl)-10-(ξ)- hydroxychrysophanol-9-anthrone showed *in vitro* antiplasmodial activity against chloroquine-sensitive 3D7 strain of *Plasmodium falciparum* and knipholone selectively inhibited leukotriene metabolism in *in vitro* a human blood assay [88]; knipholone anthrone showed *in vitro* cytotoxicity [89] and antioxidant activity on DPPH assay [90]	No previous reports

*Acmella caulirhiza* Delile (Asteraceae)	Unsaturated alkylamides like spilanthol and N-isobutylnona-2E,4E-dien-8ynamide [91] *In vitro* antiplasmodial activity [92]	Swelling [15]; tonsillitis [20, 63]; and toothache [40, 87]

*Artemisia absinthium* L. (Asteraceae)	Camphor, davanone, ethyl (E)-cinnamate, (E)-nerolidol, and chamazulene [93] Essential oils showed *in vitro* antiparasitic effects against promastigote and axenic amastigote forms of *Leishmania donovani* and *Leishmania aethiopica* and *in vitro* cytotoxicity on THP-1 (human leukaemia) cell lines [93]; and *in vitro* cytotoxicity on human leukaemia cell lines [94]	Hypertension, stomach ache, severe abdominal cramp [18] and sour throat [40]

*Artemisia afra* Jacq. ex Willd. (Asteraceae)	Epoxylinalol and dihydrocostunolide [94]; camphor, davanone, bornyl acetate, 4-terpineol, and chamazulene [95] *In vitro* cytotoxicity on human leukaemia cell lines [73]; and *in vitro* antioxidant effect on DPPH assay [95]	Stomach ache [18, 42]; evil eye [16, 17, 62]; headache [42, 77]; eye disease, tinea capitis infection, hematuria, and stabbing pain [77]; antifertility agent [33]; malaria [42, 62]; ascariasis [18]; epilepsy and febrile illness [46, 65]
*Artemisia annua* L. (Asteraceae)	*In vitro* inhibition of immune mediators of angiogenesis [96]; the sesquiterpene (Z)-7-acetoxy-methyl-11-methyl-3-methylene-dodeca-1,6,10-triene showed moderate cytotoxic activities against the human tumor cell lines of HO8910 (ovary), 95-D (lung), QGY (liver), and HeLa (cervix) by MTT assay and induced apoptosis on 95-D tumor cells [97]; artemisinin and quercetagetin 6,7,3′,4′-tetramethyl ether showed significant cytotoxicity against P-388, A-549, HT-29, MCF-7, and KB tumor cells [98]	No previous reports

*Cineraria abyssinica* Sch.Bip. ex A.Rich. (Asteraceae)	*In vitro* radical scavenging activity on DPPH assay [99]; flavonoidal glycoside (rutin) showed *in vitro* antibacterial activity [100]	No previous reports

*Guizotia scabra* (Vis.) Chiov. (Asteraceae)	*In vitro* cytotoxicity on human leukaemia cell lines [73], and *in vitro* antiviral activity [101]	Wound [20]; epilepsy [40]; and ectoparasite infestation [47]

*Solanecio gigas* (Vatke) C. Jeffrey (Asteraceae)	*In vitro* antiviral activity against human immunodeficiency virus type 1 and type 2 cytotoxicity on human T-lymphocytic MT-4 cell lines [102]	Skin diseases [62]; retained placenta [40]; hepatitis [64]; evil eye [15]

*Vernonia auriculifera* Hiern (Asteraceae)	Tannins, flavonoids, terpenoids, and saponins [103]	Toothache [72]; snake bite [42]; skin cut [47]

*Cleome brachycarpa* (Forssk.) Vahl ex DC. (Capparidaceae)	No previous reports	

*Commelina benghalensis* L. (Commelinaceae)	Phlobatannins, carbohydrates, tannins, glycosides, volatile oils, resins, balsams, flavonoids, and saponins [104] Ethanol extract showed *in vivo* sedative and anxiolytic activity [105]	Helminths [65]; skin infection [72]

*Kalanchoe petitiana* A. Rich. (Crassulaceae)	Polyphenols, alkaloids, flavonoids, tannins, saponins, and steroids [106] *In vitro* antimicrobial activity against *Escherichia coli*, *Pseudomonas aeruginosa*, and *Staphylococcus aureus* [75]; and *in vivo* wound healing activity [106]	Breast and skin cancer [107]; swelling [40, 77]; tapeworm infection, trachoma, and syphilis [77]; lymphadenopathy and evil eye [22]; sore muscles [108]; itching skin [63]; and bone fracture [23]

*Croton macrostachyus* Hochst. ex Delile (Euphorbiaceae)	Ethanol extract showed *in vitro* antioxidant activity on DPPH assay [79]	Tumor, rabies, and wart [24]; skin cancer and wound [17]; gonorrhea [20, 23, 62]; headache [18, 109]; snake bite [18, 72]; malaria [16, 18–20, 110]; helminths [18, 111]; tinea nigra [40]; ringworm [17, 62]; tinea versicolor [16, 25]; heart failure [62]; bleeding [18, 24]; hepatitis [16, 18, 24]; stomach ache [16, 18, 23]; diarrhea [16, 18]; lymph adenitis and rheumatism [18]; bloat, scabies, and urine retention [16]; retained placenta and leprosy [19]

*Euphorbia schimperiana* Scheele (Euphorbiaceae)	*In vitro* cytotoxic effect against breast cancer (MCF7), hepatocellular carcinoma (HEPG2), and cervix cancer (HELA) cells [112]	Syphilis [108]

*Albizia schimperiana* Oliv. (Fabaceae)	*In vitro* cytotoxicity on human leukaemia cells [73]	Evil eye [20]; kidney infection and liver cirrhosis [18]
*Calpurnia aurea* (Aiton) Benth. (Fabaceae)	3*β*,4*α*,13*α*-Trihydroxylupanine, calpaurine, lupinine, and epilupinine calpurmenine and calpurmenine pyrrolecarboxylic acid ester, 13-hydroxylupanine, its tiglate and pyrrolecarboxylic acid esters (calpumine), virgiline and virgiline pyrrolecarboxylic acid ester [113]; 4*β*-hydroxy-13*α*-*O*-(2′-pyrrolylcarbonyl)-lupanine (digittine) and 4*β*,13*α*-dihydroxylupanine [114]; alkaloids, tannins, flavonoids, and saponins [35] Methanol extract showed *in vitro* antimicrobial activity against *Staphylococcus aureus*, *Escherichia coli*, and *Pseudomonas aeruginosa* [75] and type 1 and type 2 human immunodeficiency virus and showed cytotoxicity on human T-lymphocytic MT-4 cell lines [102]; methanol and dichloromethane crude extracts showed *in vitro* cytotoxicity on human leukaemia cells [73]; and ethanol extracts showed *in vitro* antioxidant activity on DPPH assay [79]	Tumor [22, 26, 80]; stomach ache [21, 62, 70, 81]; wound and skin infection [62]; Gonorrhoea and syphilis [16], amoebiasis [16, 80]; ascariasis and gastric ulcer [23]; diarrhea [21, 38, 70]; scabies and pubic hair louse [40]; diabetes mellitus and hypertension [19]; herpes zoster, hemorrhoids and tinea capitis [21]; and swelling and tuberculosis [58]

*Crotalaria agatiflora* Schweinf. (Fabaceae)	Methanol and dichloromethane crude extracts showed *in vitro* cytotoxicity on human leukaemia cells [73]	No previous reports
*Crotalaria incana* L. (Fabaceae)	Dihydrosenecionine isomer, nemorensine isomer, integerrimine and anacrotine [115] Methanol and dichloromethane crude extracts showed *in vitro* cytotoxicity on human leukaemia cell lines [73]	

*Senna singueana* (Delile) Lock (Fabaceae)	Methanol extracts showed *in vitro* antioxidant activity on DPPH assay [116]	Stomach ache [58, 62, 70]; wound and swellings [62]; teeth infection and sprain [58]

*Ajuga leucantha* Lukhoba (Lamiaceae)	No previous reports	Diarrhea [70]

*Leonotis ocymifolia* (Burm.f.) Iwarsson (Lamiaceae)	Methanol and dichloromethane crude extracts showed *in vitro* cytotoxicity on human leukaemia cells [73]	Ascariasis [62], febrile illness [16, 62]; eye disease [16]; headache and neck ulcer [55]; and snake bite [15]

*Ocimum gratissimum* L. (Lamiaceae)	Essential oil contains constitutes *γ*-terpinene, *β*-phellandrene, limonene, and thymol and showed *in vivo* antiplasmodial activity against *Plasmodium berghei* infection [117]	Allergy reaction [18, 20]; rheumatism, headache and eye disease [18]; febrile illness and general malaise [40]; sun stroke [24]; malaria [44]

*Pycnostachys abyssinica* Fresen. (Lamiaceae)	No previous reports	Eye disease [18, 47]; ascariasis and wound [18]; diarrhea, stomach ache, amoebiasis, stomach bloating, and food poisoning [70]; headache [63]

*Salvia nilotica* Juss. ex Jacq. (Lamiaceae)	Essential oil contains germacrene D, guaiol, and *trans*-caryophyllene as major constituents and showed activity against both Gram-positive and Gram-negative pathogenic bacteria; the oil also showed *in vitro* antioxidant activity on DPPH assay [118]	Tonsillitis and constipation [62]; herpes simplex [18, 38]; wound [40]; lymphadenitis [63]; and hemorrhoids and diarrhea [65]

*Thymus schimperi* Ronniger (Lamiaceae)	Phenol and flavonoid compounds, and aqueous methanol extract showed *in vitro* radical scavenging ability, iron reducing power, and total antioxidant capacity [119]	Diabetes [62]; hypertension [18, 40]; tonsillitis [18]; toothache [18, 21]; abdominal pain [21]; and cough [38, 55]

*Sida schimperiana* Hochst. ex A. Rich. (Malvaceae)	No previous reports	“Shotelaye” (hydrops fetalis) [21, 22]; cough and fever [62]; diarrhea [18]; wound [25, 62]; bleeding and evil eye [24]; glandular disease and rabies [40]; amoebic dysentery, and liver disease [65]; paralysis [21]; epilepsy [43]
*Bersama abyssinica* Fresen. (Melianthaceae)	Flavonol glycosides isoquercetrin, hyperoside, quercetin-3-*O*-arabinopyranoside, kaempferol-3-O-arabinopyranoside, xanthone glycoside, mangiferin [115] Ethanol water extracts showed *in vitro* antioxidant activity on DPPH assay and antiproliferative activity on human liver carcinoma cell line and normal human fetal lung cells [79]; methanol extract showed *in vitro* antioxidant activity on DPPH assay [115], and antiviral activity against type 1 human immunodeficiency virus [102]	Tumor, dysentery and roundworms [107, 109]; ascariasis [15, 38, 81, 109]; wound [20]; stomach ache [17]; snake bite and liver diseases [70]; tonsillitis [72]; bronchitis and febrile illness [42, 43]

*Dorstenia barnimiana* Schweinf. (Moraceae)	Phytochemical screening showed the presence of coumarins [34]	Cancer [26]; hepatitis, syphilis and rabies [25, 26]; skin cancer, dysentery, wart and fever [25]; pulmonary tuberculosis, leprosy, and stomach illness [22]

*Syzygium guineense* (Willd.) DC. (Myrtaceae)	Methanol and dichloromethane crude extracts showed *in vitro* cytotoxicity on human leukaemia cells [73] and antimicrobial activity [120]	Stomach ache [17–19, 23]; diarrhea [15, 18, 19, 24], kidney infection, liver cirrhosis, and tonsillitis [18]; syphilis [23, 80]; malaria, hemorrhoid, internal worms, snake bite, and gonorrhea [65]

*Oxalis corniculata* L. (Oxalidaceae)	*In vivo* antitumor activity against Ehrlich ascites carcinoma on mice [121]	Wound [17]; arthritis [63]; tape worm infection [21]

*Rumex nervosus* Vahl (Polygonaceae)	Alkaloids, flavonoids, terpenoids, tannins, glycosides, and volatile oils [122]	Breast cancer, gastritis, and snake bite [16]; wart [15, 22]; hepatitis [49, 55]; skin rash [16, 21]; bleeding [15, 40, 81, 109]; wound [40, 49, 55, 62, 109, 110]; scabies and acne vulgaris [62]; ascariasis and herpes simplex [21]; stomach ache and dysentery [22]; diarrhea [49]; eye problems and round worm [55]

*Rumex nepalensis* Spreng. (Polygonaceae)	Anthraquinones, naphthalenes, tannins, stilbenoids [123] Ethanol water extracts showed *in vitro* antiproliferative activity on human liver carcinoma cell line and on normal human fetal lung cells and antioxidant activity on DPPH assay [79], and methanol and dichloromethane crude extracts showed *in vitro* cytotoxicity on human leukaemia cells [73]	Wound, ascariasis, abdominal bleeding, gastric ulcer, and hemorrhage [23, 80]; gastritis [18]; stomach problems [108]; leishmaniasis [25]; abdominal cramp and ear infection [63]; tonsillitis [18, 25]

*Clematis simensis* Fresen. (Ranunculaceae)	Triterpenoids, saponins, alkaloids, polyphenols, and unsaturated sterols [120] *In vivo* anti-inflammatory and antinociceptive activities [124]	Cancer and hemorrhoid [15]; wart and evil eye [24, 40]; wound [15, 24, 40, 63, 81]; tonsillitis [62]; eye infection [63]; leg swelling, malaria, and mental illness [49]; stomach ache [47]

*Prunus africana* (Hook.f.) Kalkman (Rosaceae)	No previous reports	Benign prostatic hyperplasia and prostate gland hypertrophy [20]; cancer, respiratory disorders, bad breathe, diarrhea, gonorrhea, tuberculosis, and ear problems [22]; swelling [40]; wounds [19, 22]; tonsillitis [23, 80]

*Clausena anisata* (Willd.) Hook.f. ex Benth. (Rutaceae)	Carbazole alkaloids, peptide derivatives, sitosterol, and stigmasterol [125] Methanol and dichloromethane crude extracts showed *in vitro* cytotoxicity on human leukaemia cells [73]	Skin irritation [20]; toothache [40]; ascariasis [19]; evil eye [24, 25, 63]

*Osyris quadripartita* Salzm. ex Decne. (Santalaceae)	Alkaloids, phenols, terpenoids, tannins, saponins, and flavonoids [126] Methanol extracts showed *in vitro* antimicrobial activity against *Escherichia coli*, *Pseudomonas aeruginosa, Staphylococcus aureus*, *Candida albicans*, and *Trichophyton mentagrophytes* [11]; *in vitro* inhibition of NO production and cytotoxicity against MCF-7 and NCI-H460 cell lines [127]	Cancer [62]; anaphylactic shock, evil eye, and epilepsy [18]; eczema [40]; toothache [46]
*Dodonaea viscosa* subsp. *angustifolia* (L.f.) J.G.West (Sapindaceae)	Alkaloids, terpenoids, saponins, tannins, sugars, phenolics, and flavonoids [128] Methanol extracts showed *in vivo* nonsensitizer effect in mice using the mouse ear swelling test method [129], *in vitro* antiviral effect against type 1 human immunodeficiency virus [102], and *in vitro* free radical scavenging activity on DPPH assay [128]	Malaria [57]

*Brucea antidysenterica* J.F.Mill. (Simaroubaceae)	Flavonoids, amino acids, and vitamin C [130] *In vitro* antiplasmodial activity against *Plasmodium berghei* infection [131]	Cancer/tumor [107]; wart [24]; rabies [18, 62]; leprosy [62]

*Discopodium penninervium* Hochst. (Solanaceae)	5*α*,17*β*-Dihydroxy-6*α*,7*α*-epoxy-1-oxowitha-2,24-dienolide, withanone, and withanolide A [132], 5,6-epoxy-16-oxygenated withanolides, jaborosalactone-L, and 17-epiacnistin-A [133, 134]; 6*α*,7*α*-epoxy-1-oxo-5*α*,12*α*,17*α*-trihydroxywitha-2,24-dienolide and a coloratane sesquiterpene, 7*α*,11*α*-dihydroxy-4(13),8-coloratadien-12,11-olide, withanone, 5*α*,17*β*-dihydroxy-6*α*,7*α*-epoxy-1-oxowitha-2,24-dienolide, 7*α*,11*α*-dihydroxy-8-drimen-12,11-olide, withasomnine, and (E,Z)-9-hydroxyoctadeca-10,12-dienoic acid [135] Jaborosalactone-L showed cytotoxicity only to the murine macrophage cell line, RAW 264.7, but the 16*α*-oxygenated withanolides exhibited cytotoxicity to both human (COR-L23 and ECV 304) and murine (L929 and RAW 264.7) carcinoma cell lines with IC_50_ values ranging from 1.2 to 150 *μ*M [136]. 6*α*,7*α*-Epoxy-1-oxo-5*α*,12*α*,17*α*-trihydroxy-witha-2,24-dienolide inhibited COX-2 and LTB4 formation; 7*α*,11*α*-dihydroxy-4(13),8-coloratadien-12,11-olide and withasomnine inhibited LTB_4_ biosynthesis but showed minor inhibition of COX-1 and COX-2 [135]	Skin detoxification [62]; and liver disease [70]

*Gnidia involucrata* Steud. ex A.Rich. (Thymelaeaceae)	Flavonoids and glycosides [137]	Ascariasis, evil eye, anthrax, intestinal helminths, and gland swelling [18]

*Lantana trifolia* L. (Verbenaceae)	Flavone glycosides (scutellarein-*7-O*-*β*-D-apiofuranoside and apigenin-*7-O*-*β*-D-apiofuranosyl-(1⟶2)-*β*-D-apiofuranoside) and the flavone celtidifoline (5,6,40,50-tetrahydroxy-7,30-dimethoxyflavone) [138, 139]	Headache [70]; malaria [71]

*Lippia adoensis* Hochst. (Verbenaceae)	Limonene, perillaldehyde, piperitenone, and 2-methyl-6-methylene-2,7-octadien-4-one [140], sesquiterpene hydrocarbon (germacrene D) [141] Methanol extract showed *in vitro* cytotoxicity on human leukaemia cell lines [73], and antimicrobial activity against *Staphylococcus aureus*, *Escherichia coli*, and *Pseudomonas aeruginosa* [75]; water extracts showed *in vivo* protection/relieve against acetic acid induced writhing in mice model [142]	Eczema, fungal infections, common cold, and cough [62]; intestine swelling [18]; gastrointestinal disorder [40]; abdominal irritation and acute stomach illness [46]

*Cyphostemma serpens* (Hochst. ex A.Rich.) Desc. (Vitaceae)	No previous reports	

**Table 5:** Informants consensus factor for different ailment categories.

No.	Category	No. of species	% of all species	No. of use reports	% of all use reports	ICF
1	Skin	25	30.5	32	26.5	0.23
2	Breast	20	24.4	29	23.9	0.32
3	Cervical	1	1.22	1	0.83	0
4	Colon	1	1.22	1	0.83	0
5	Lung	1	1.22	1	0.83	0
6	Throat	1	1.22	1	0.83	0
7	Other disease	33	40.2	56	46.3	0.42
	*Total*	*82* ^*∗*^		*121*		

^*∗*^Each plant species may be listed in several categories.

**Table 6:** Summary of stats for relative frequency of citation (RFC) and cultural importance index (CI).

	Mean	Standard deviation	Minimum	Maximum

UV	1.8	1.1	0.5	6
RFC	0.02	0.015	0.014	0.1
CI	0.034	0.027	0.014	0.16

Association between RFC and CI by using Pearson correlation method				
	UV	RFC	CI	

UV	1			
RFC	−0.36^*∗*^	1		
CI	0.003	0.858^*∗∗*^	1	

^*∗*^Correlation is significant at 0.05 level. ^*∗∗*^Correlation is significant at 0.001 level.

## Data Availability

The authors declare that all data supporting the finding of this study are included in this article and its supplementary information files.
